# Aluminum Plasmonics Enriched Ultraviolet GaN Photodetector with Ultrahigh Responsivity, Detectivity, and Broad Bandwidth

**DOI:** 10.1002/advs.202002274

**Published:** 2020-11-17

**Authors:** Abhishek Dubey, Ragini Mishra, Yu‐Hung Hsieh, Chang‐Wei Cheng, Bao‐Hsien Wu, Lih‐Juann Chen, Shangjr Gwo, Ta‐Jen Yen

**Affiliations:** ^1^ Department of Materials Science and Engineering National Tsing Hua University Hsinchu 300 Taiwan; ^2^ Institute of NanoEngineering and MicroSystems National Tsing Hua University Hsinchu 300 Taiwan; ^3^ Research Centre for Applied Science Academia Sinica Taipei 115‐29 Taiwan; ^4^ Department of Physics National Tsing Hua University Hsinchu 300 Taiwan

**Keywords:** epitaxial aluminum film, GaN, UV photodetection, UV Plasmonics

## Abstract

Plasmonics have been well investigated on photodetectors, particularly in IR and visible regimes. However, for a wide range of ultraviolet (UV) applications, plasmonics remain unavailable mainly because of the constrained optical properties of applicable plasmonic materials in the UV regime. Therefore, an epitaxial single‐crystalline aluminum (Al) film, an abundant metal with high plasma frequency and low intrinsic loss is fabricated, on a wide bandgap semiconductive gallium nitride (GaN) to form a UV photodetector. By deliberately designing a periodic nanohole array in this Al film, localized surface plasmon resonance and extraordinary transmission are enabled; hence, the maximum responsivity (670 A W^−1^) and highest detectivity (1.48 × 10^15^ cm Hz^1/2^ W^−1^) is obtained at the resonance wavelength of 355 nm. In addition, owing to coupling among nanoholes, the bandwidth expands substantially, encompassing the entire UV range. Finally, a Schottky contact is formed between the single‐crystalline Al nanohole array and the GaN substrate, resulting in a fast temporal response with a rise time of 51 ms and a fall time of 197 ms. To the best knowledge, the presented detectivity is the highest compared with those of other reported GaN photodetectors.

## Graphical abstract

Aluminum plasmonics is merged as an advancement tool to boost GaN a superior complementary metal oxide semiconductor (CMOS) compatible UV photodetector. By introducing a single‐crystalline Al nanohole array on a GaN substrate, it enables ultraviolet plasmons and Schottky barrier, and thus demonstrates excellent performance in entire UV regime: maximum responsivity (670 A W^−1^), highest detectivity (1.48 × 10^15 ^cm Hz^1/2^ W^−1^), and fast temporal response.

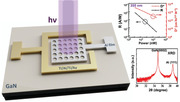

Recently, plasmonics have been utilized in numerous applications, such as photovoltaics,^[^ ^1^, ^2^ ^]^ water splitting,^[^ ^3^ ^]^ nonlinear optics,^[^ ^4^ ^]^ surface‐enhanced Raman scattering,^[^ ^5^ ^]^ plasmonic lasing,^[^ ^6^ ^]^ gas sensing^[^ ^7^ ^]^ and solar cells.^[^ ^8^ ^]^ More recently, researchers have further benefited from plasmon‐enhanced light–matter interactions to advance the applications of photodetection.^[^ ^9^, ^10^, ^11^, ^12^ ^]^ Thus far, the most typically used materials for plasmonic applications are silver (Ag) and gold (Au), mainly owing to their low intrinsic loss. Nevertheless, Ag and Au are affected by interband transitions at 300 nm^[^ ^13^ ^]^ and 500 nm,^[^ ^14^ ^]^ respectively. Therefore, plasmonics‐empowered photodetections have been achieved up to IR and visible frequencies, but rarely in the UV regime.^[^ ^15^, ^16^, ^17^ ^]^ Instead of Ag and Au, aluminum (Al), the most abundant metal in the Earth's crust, can fulfill the need for less investigated UV plasmonics. Al is advantageous owing to its low intrinsic loss, high plasma frequency (≈15 eV), low screening (*ε*_∞_ ≈ 1), and complementary metal oxide semiconductor (CMOS) compatibility.^[^ ^18^, ^19^, ^20^, ^21^ ^]^

The demand for UV photodetection is currently increasing in various fields, such as medical science, civilian and defense regions, biological detection, UV astronomy, and endo‐atmospheric sensing.^[^ ^22^, ^23^, ^24^, ^25^, ^26^, ^27^, ^28^ ^]^ Combining Al plasmonics with GaN is an excellent method for various applications, such as ultrahigh responsivity, detectivity, and visible blind photodetection. Recently, Ahmadivand et al. theoretically demonstrated the use of Al oligomers on GaN for ultraviolet photodetection;^[^ ^29^ ^]^ however, experimental studies of Al plasmonics with GaN have not been performed.

Herein, we present a UV plasmonics‐empowered UV photodetector comprising a single‐crystalline Al nanohole array on an undoped GaN/Al_2_O_3_ substrate. These Al periodic nanoholes are designed to enable localized surface plasmon resonance (LSPR), which then decays nonradiatively and penetrates into the GaN substrate through a Schottky junction. In addition, these nanoholes support extraordinary transmissions, which facilitate the coupling of incident illumination to the GaN surface more efficiently to excite more photoelectrons. Evidenced through both FDTD simulations and micro UV reflectance measurements, such a UV plasmon‐assisted photodetector demonstrated ultrahigh sensitivity, ultrahigh responsivity, CMOS compatibility, and visible blindness in the demanded UV regime.

The single‐crystalline Al film was grown via plasma‐assisted molecular beam epitaxy (PA‐MBE) on undoped GaN/Al_2_O_3_ substrates. We monitored the in‐situ growth by employing streaky reflection high‐energy electron diffraction patterns and observed the excellent crystallinity of the atomically epitaxial and smooth Al film (see Figure S1 in the Supporting Information). In addition, we further scrutinized the crystallinity, lattice orientation/coherence, and surface roughness by performing X‐ray diffraction (XRD), transmission electron microscopy, and atomic force microscopy (AFM) analyses, respectively. For example, as shown in **Figure** **1**a, the two peaks at 34.8° and 38.7° in the XRD pattern correspond to the (0002) plane of GaN and the (111) plane of Al, respectively, confirming the single crystallinity and the primary (111) crystal orientation of the as‐grown Al film.

**Figure 1:**
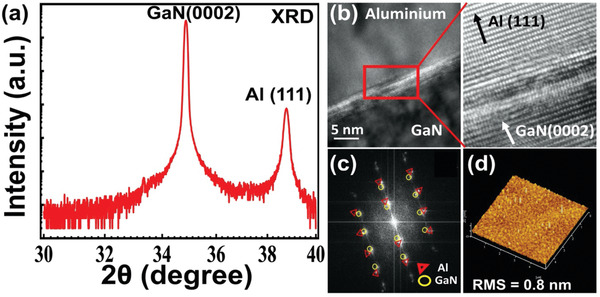
Ex‐situ characterization of single‐crystalline Al film a) X‐ray diffraction (XRD) measurement of single‐crystalline Al film on GaN/Al_2_O_3_ shows the XRD peak of GaN (0002) and Al (111) peak at 34.8° and 38.7° respectively. b) Interface Transmission electron microscope (TEM) image shows atomic arrangement of single crystalline Al (111) film on GaN (0002) substrate. c) SAED pattern of Al and GaN/Al_2_O_3_ at interface shows the aligned (0002) GaN plane with (111) Al plane. d) Atomic force microscopy (AFM) image (area: 5 µm × 5 µm) shows surface roughness of epitaxial single crystalline aluminum film on GaN substrate.

Furthermore, the atomic arrangement of the metal (Al) and semiconductor (GaN) interface (M/S) is shown in Figure 1b. At the interface, each Al atom was attached to Ga atoms owing to the induced strain. After strain relaxation in the (111) plane of Al due to dislocation and misfit, 11 Al atoms were interconnected with 10 Ga atoms. The lattice mismatch between the Al and GaN films was calculated to be 10%. The SAED pattern in Figure 1c shows the sustained effect of the aligned Al and GaN M/S interfaces. Furthermore, the smoothness of the Al film was confirmed by measuring the root mean square (RMS) roughness using AFM. As shown in Figure 1d, a 5 µm × 5 µm AFM image indicated a 0.8 nm RMS roughness. Based on the single‐crystalline Al film on the GaN/Al_2_O_3_ substrate, we fabricated an Al nanohole array measuring 100 µm × 100 µm, which enabled photodetection in the UV regime by plasmonic effects. The fabrication process of this plasmonics‐empowered photodetector is presented in **Figure** **2**a, and the important details are explained in the experimental section.

**Figure 2:**
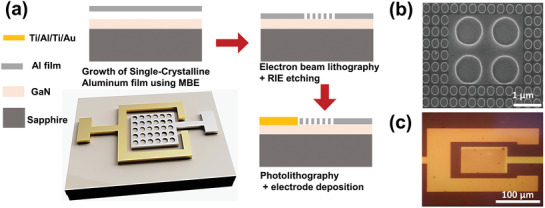
Device fabrication flow a) Schematic and fabrication process of Al nanohole array and UV plasmons ‐empowered photodetector using electron beam lithography, TCP coupled RIE etching, photolithography, and ohmic contact deposition. b) Scanning electron microscopy (SEM) image of periodic Al nanohole array with 220 nm diameter and 320 nm periodicity. c) Optical microscope image of as fabricated device.

We optimized the dimensions of the Al nanoholes for exciting LSPR and for achieving the maximum extraordinary transmission (EOT) at the GaN surface^[^ ^30^ ^]^ simultaneously. The optimized periodicity and diameter were 320 and 220 nm, respectively, which matched the wavelength of the LSPR with those of the external illumination and near the bandgap edge of GaN (i.e., 355 nm). The thickness of the Al nanoholes (i.e., 65 nm) was optimized to achieve the maximum EOT at the LSPR wavelength using FDTD simulations (See Figure S3 in the Supporting Information). First, we obtained the LSPR dipole mode at 355 nm, as indicated by both the experimental and simulation reflectance spectra shown in **Figure** **3**a. This LSPR dipole mode enabled an EOT and hence increased the stimulation of GaN photoelectrons. In addition to the aforementioned LSPR mode, another mode appeared in the shorter wavelength regime. This additional mode appeared owing to the coupling among nanoholes, which facilitates the broadening of the range of greater transmittance for practical applications. Finally, we analyzed the electric field confinement (|*E*|^2^) [at the top edge of the holes and the cross‐sectional direction] under a normal incident illumination at a 355 nm wavelength. As shown in Figure 3b,c, the *E_z_* component manifested field confinement from the induced electric dipole around the top edge, thereby promising higher photodetection responses.

**Figure 3:**
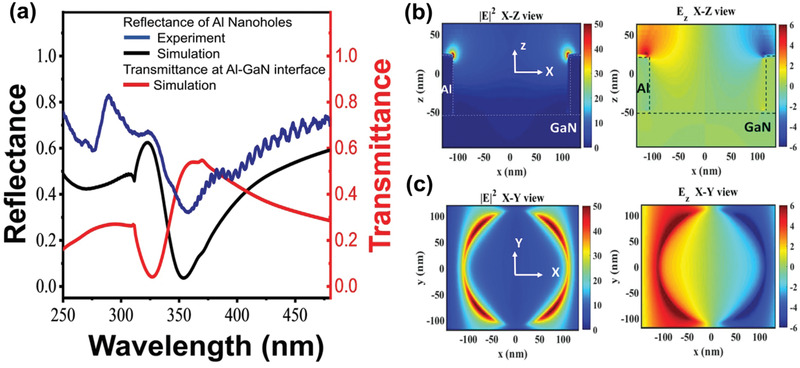
a) Experimental reflectance measurement of Al nanohole array measured by micro UV reflectance optical setup (blue curve) shows the LSPR resonance dip at 355 nm (illumination laser wavelength) well match with simulated (FDTD) reflectance measurement (black curve) and simulated (FDTD) transmittance at Al and GaN interface (red curve) to show EOT phenomena to activate the photoelectron from GaN. The 355 nm plasmonic resonance of nanohole array is chosen to match with excitation wavelength and near band gap edge of GaN. b,c) FDTD simulated Electric field enhancement analysis plots of the Al nanohole in *XZ* and *XY* view, (∣*E*∣^2^) and *E_z_* confinement of nanohole show the electric field confinement and formation of resonant electric dipole at the edges of nanohole at 355 nm wavelength.

To demonstrate the ultrahigh response and detectivity of the fabricated photodetector, we conducted various optoelectrical characterizations. The first was to characterize the dark and illuminated current–voltage (*I*–*V*) relationship, in which we illuminated the as‐fabricated UV plasmons empowered photodetector via a solid‐state continuous wave laser at the resonance wavelength of 355 nm to maximize the number of generated UV plasmons and the excited photocarriers simultaneously. The corresponding results are shown in **Figure** **4**a. In the reverse bias region, the dark current was extremely low (less than 10^−6^ A), confirming the Schottky nature.^[^ ^31^ ^]^ Such a low dark current originated from the released strain at the epitaxial interface between the Al (111) plane and the GaN (0002) plane; it induced an additional piezoelectric force‐based electric field to increase the Schottky barrier height beyond that achieved by conventional thermal evaporation deposition.^[^ ^32^ ^]^ We calculated the Schottky barrier height by applying the thermionic emission model,^[^ ^33^ ^]^ which was 0.60 eV at the Al–GaN interface, in contrast to the well‐known Ohmic contact of the thermally deposited Ti/Al–GaN interface. In addition, we regulated the incident optical power from 11 nW, 155 nW, 657 nW, and 2.5 µW to 31.5 µW to characterize the behavior of this photodetector. We observed the intensive enhancement of photocurrents in the reverse bias mode at these low powers.

**Figure 4:**
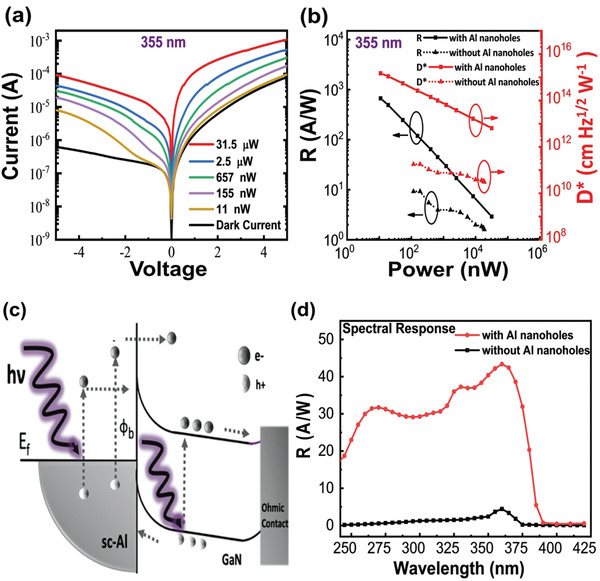
Photoresponse characteristics a) *I*–*V* measurement of UV plasmons empowered photodetector in dark and using plasmonic resonance matched 355 nm laser with different illuminated optical power (11 nW, 155 nW, 657 nW, 2.5 µW, and 31.5 µW). b) Calculated Responsivity (*R*) and detectivity (D*) as function of 355 nm laser illuminated power. The maximum detectivity and responsivity 1.48 × 10^15 ^cm Hz^1/2^ W^−1^ and 670 A W^−1^ are achieved at 5 V reverse bias with Al nanohole array. c) schematic energy band alignment of photodetector at reverse bias. d) Spectral responsivity spectra of as fabricated photodetector with and without Al nanohole array from deep UV to near UV regime. This confirms the broad bandwidth nature due to excitation of higher modes in the Al nanohole array.

In the second optoelectrical characterization, both the responsivity (*R*) and detectivity (D*) were analyzed. These two parameters are the figures of merit (FoM) to determine the capacity of photodetectors,^[^ ^34^, ^35^, ^36^ ^]^ and we evaluated them at a reverse bias of 5 V to determine the maximum effect of the device. *R* and D* are expressed by Equations (1) and (2), respectively, as follows

(1) $$\begin{array}{c} R=\frac{\Delta I\;\left(A\right)}{P\;\left(W\right)} \end{array}$$

(2) $$\begin{array}{c} D^{*}=\frac{R\sqrt{A}}{\sqrt{2eI_{\mathrm{dark}}}}\; \end{array}$$

where Δ*I* = (*I*_light_ − *I*_dark_), I_light_ and I_dark_ are the photocurrents with and without illumination in Amperes (A), respectively. P is the total illumination optical power in the active device area (i.e., 100 µm × 100 µm) in Watts (W). A is the active area and e is the charge of an electron. Figure 4b shows the power‐dependent *R* and D* of the photodetector with and without the Al nanohole array illuminated with a 355 nm laser at a 5 V reverse bias. We observed the maximum *R* of 670 A W^−1^ at an 11 nW illumination with the Al nanohole array; in addition, the measured D*, an FoM determining the minimum illumination power detectable by the photodetector, indicated the maximum value of 1.48 × 10^15 ^cm Hz^1/2^ W^−1^. Therefore, by using our optimized Al nanohole array at 355 nm incident light, we observed almost 73 times and 8.2 × 10^3^ times excellent enhancement in responsivity and detectivity, respectively. Thus far, the measured D* is the highest among all types of GaN‐based UV photodetectors, as shown in **Table** **1**. To demonstrate these ultrahigh *R* and D*, we investigated the three key factors driven by Al plasmonics: epitaxial growth of single‐crystalline Al film for exciting stronger UV plasmons, optimization of the Al nanohole array for increasing the EOT at the GaN interface, and formation of a Schottky junction between Al and GaN for harvesting photocharges, as presented in Figure 4c.^[^ ^37^ ^]^

**Table 1:** Figure of merits of state of art GaN based UV photodetectors

Device	Responsivity [A W^−1^]	Detectivity [cm Hz^1/2^ W^−1^]	*t*_r_ [s]	*t*_f_ [s]	Ref.
GaN/Ag NPs	4	–	–	–	^[^ ^15^ ^]^
GaN/Au NPs	11	–	2.9	6.2	^[^ ^39^ ^]^
Ni/GaN/Au	1.31	–	–	–	^[^ ^43^ ^]^
Ni/GaN/Ti/Al	0.104	–	–	–	^[^ ^44^ ^]^
GaN NWs	0.47	–	–	–	^[^ ^42^ ^]^
GaN(p–i–n)	0.23	–	–	–	^[^ ^41^ ^]^
GaN‐Thin film	13.02	–	0.21	1.2	^[^ ^40^ ^]^
GaN micro Wire	450	2.08 × 10^11^	0.07	0.09	^[^ ^45^ ^]^
GaN Mesoporous	10^4^	5.3 × 10^14^	20	60	^[^ ^46^ ^]^
a‐GaN film	400	6.6 × 10^12^	0.173	1.21	^[^ ^47^ ^]^
GaN‐phototronic	0.03	1.78 × 10^12^	0.1	0.1	^[^ ^37^ ^]^
Al Plasmonics based‐GaN	670	1.48 × 10^15^	0.051	0.197	This Work

In addition to a 355 nm laser, our photodetector can be used for other UV frequencies because LSPR validates a broad spectral coverage.^[^ ^38^ ^]^ For example, we evaluated this plasmon‐empowered UV photodetector using a 325 nm laser. The maximum *R* and D* were 307 A W^−1^ and 7 × 10^12^ cm Hz^1/2^ W^−1^, respectively, as shown in Figure S5 in the Supporting Information. Based on our knowledge, this is the maximum detectivity at both 355 and 325 nm, which are the highest values reported to date in GaN‐based photodetectors (see Table 1). Moreover, the spectral photoresponsivity of the photodetector with and without Al nanohole array are shown in Figure 4d. The plotted photoresponsivity in the UV regime reveals a significant enhancement through the entire band, in contrast to that without the Al nanohole array, even up to unusual deep UV frequencies. In this study, the measured spectral photoresponse spectra agreed well with the experimentally measured Al nanohole array reflectance spectra, as shown in Figure 3a. Such broad spectral coverage and spectral response peak at approximately 270 and 325 nm, respectively, are attributed to the cross talking and excited higher order resonance modes of the single‐crystalline Al nanohole array. Optimization of the diameter of these nanoholes excite not only higher resonance modes, but also plasmonic coupling among nanoholes to broaden the spectral photo enhancement. In addition, we performed photoresponse behavior in the deep UV regime by using a 266 nm laser. As expected, there also appeared significant photoresponse, as shown in Figure S6 in the Supporting Information.

The third optoelectrical characterization was temporal photoresponse analysis, which is essential for commercializing a photodetector. As presented in **Figure** **5**a, with a 355 nm illumination and a 5V reverse bias, we observed that the plasmon‐empowered UV photodetector exhibited excellent and stable transient photocurrents for various low optical powers of 27, 700, 15, and 30 µW. Notably, such excellent and stable performances were also demonstrated at 325 nm, verifying the broadband application in the UV regime (see Figures S4 and S5 in the Supporting Information). In accordance with the measured temporal photocurrent response, we further assessed the rising and falling times of this UV photodetector. As shown in Figure 5b, a rise time of 51 ms and a fall time of 197 ms were shown under UV illumination which are very much faster in comparison to GaN photodetectors without the Al nanohole array (see Figure S4b in the Supporting information). In addition, the exponential decay behavior of photocurrent and large difference in rising and falling time is attributed to the persistent photoconductive behavior of GaN.^[^ ^48^ ^]^ Based on our knowledge, this temporal photoresponse that outperformed those reported plasmonics‐designed GaN photodetectors as shown in the Table 1.

**Figure 5:**
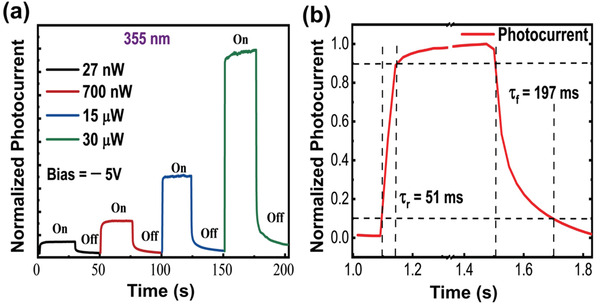
Temporal photoresponse a) Stable transient photocurrent measurement of UV photodetector using 355 nm laser illumination with different illuminated optical power (27 nW, 700 nW, 15 µW, and 30 µW) at reverse bias 5 V. b) Calculation of rising and falling time of photodetector for 355 nm laser excitation. 51 and 197 ms, rising and falling time are calculated, 10% and 90% minimum and maximum photocurrent are accounted to calculate rising and falling time.

In this study, by employing Al plasmonics on a wide bandgap semiconductor (GaN), superior photodetection was achieved in the unusual UV regime. We designed and fabricated a single‐crystalline Al nanohole array, generating the synergetic effects of LSPR, EOT, and Schottky junction. First, the LSPR and EOT were confirmed by micro UV reflectance measurements and FDTD simulation, where a significant plasmonic resonance of Al nanoholes at 355 nm matching that near the bandgap edge of GaN was indicated. Next, based on the integration of the single‐crystalline Al nanohole array and the GaN substrate, the resulting UV photodetector revealed the maximum *R* of 670 A W^−1^ and the highest D* of 1.48 × 10^15 ^cm Hz^1/2^ W^−1^. In addition, we observed that these spectral photoresponses encompassed the entire UV regime. Such an excellent broadband behavior was achieved by optimizing the periodicity and diameter of the Al nanoholes to introduce coupling among the plasmonic structures. Finally, the Al and GaN formed a Schottky junction, which not only offered intensive and stable transient photocurrents, but also enabled the fastest temporal photoresponses with a rise time of 51 ms and a fall time of 197 ms. Based on these unprecedented merits of ultrahigh *R*, D*, broad bandwidth, fast temporal response, and CMOS compatibility, this plasmonics‐empowered UV photodetector outperformed other reported plasmonics‐designed GaN photodetectors, thereby providing a basis for practical applications in defense, UV astronomy, biological, and medical sciences.

## Experimental Section

##### Growth of single‐crystalline Al Film and Fabrication of Photodetector

DCA‐600, plasma‐assisted molecular beam epitaxy was used to grow 65 nm thick single‐crystalline Al film on commercial procured 2inch undoped GaN/Al_2_O_3_ wafer. First, the substrate was thermally annealed at 800 °C for 3 h. A 100 cc Kundsen cell of Al was heated to grow 65 nm thick single‐crystalline Al at 140 nm h^−1^ deposition rate. However, the substrate was kept at room temperature (≈300 K). Before fabrication, the sample was cleaned by ethanol, IPA, and DI water for 10 min. Positive PMMA photoresist was used in the electron beam lithography (ELS 7500). To open the nanoholes, Transformer coupled plasma (TCP by LAM research) dry etching technique was used. For etching in TCP, Cl_2_ and BCl_3_ gases with 400 W RF power and 125 W bias power were used. After etching, the photoresist is cleaned by oxygen plasma cleaning. Later, photolithography and TCP dry etching were performed to pattern the Al in the Schottky electrode form. For electrode deposition, due to small sample size, image reversal photolithography was used to design ohmic contact and Ti/Al/Ti/Au (15/45/5/30 nm) contacts were deposited by e‐gun deposition technique. AZ 5214E photoresist was used for photolithography steps. The distance between the two electrodes was kept 20 µm.

Modelling was performed using the finite difference time domain method (FDTD, Lumerical Inc.). A 3D simulation domain with periodic boundary conditions were used to simulate periodic nanoholes defined by a diameter D (≈220 nm) and depth H (≈65 nm). A 3.5 nm natural oxide layer thickness was also considered on top of Al. Due to periodicity, only one nanohole was simulated. The experimentally measured single‐crystalline Al film's refractive index values by ellipsometer were used to simulate the Al nanohole structure. A plane wave source was defined to inject the plane wave from above the nanohole. A rectangular monitor was placed behind the source to measure reflectance and one monitor was placed on the interface of Al nanohole and GaN to know the extraordinary transmission at GaN surface. The electric field distribution monitor was kept on just 5 nm above the nanohole in *XY* view and for *XZ* view it was placed in the center of the nanohole. The mesh size was set to be 1 nm in *x*, *y*, and *z* directions. The reflectance and transmittance spectra were recorded between 250 nm and 500 nm.

The reflectance measurement was performed using in‐house micro reflectance setup. A broadband laser‐driven light source (LDLS EQ‐99X) from 190 to 2100 nm was used. All Plano convex lens and beam splitter were silica fused UV‐Visible compatible with 240–500 nm range. For Objective lens, Thor lab designed achromatic micro‐spot UV focusing (LMU‐40X‐UVB) objective lens with 0.50 NA was used. The reflected light signals were collected thorough andor solis SR‐500 spectrometer.

For optoelectronic characteristic, photocurrent was measured using Keithley 4200 SCS semiconductor parameter analyzer and CW 355 nm diode laser, He‐Cd 325 nm laser, and FQCW 266 nm laser. The laser was focused in the microscope imaging system on the Al nanohole array, to provide effect only form metasurface combined GaN. For spectral photoresponse, micro‐Horiba (grating 2440/500) monochromator with LDLS light source was used. The light spot was narrowed up to 100 µm to focus through a reflective objective lens (LMU‐40X‐UVB) on the Al nanhole array as a photocurrent measurement microscope setup. New port model 842‐PE power meter was used to measure the optical power of incident light.

## Conflict of Interest

The authors declare no conflict of interest.

## Supporting information

Supporting Information
