# Supplementary material for: Aluminum Plasmonics Enriched Ultraviolet GaN Photodetector with Ultrahigh Responsivity, Detectivity, and Broad Bandwidth
Source: Adv Sci (Weinh). 2020 Nov 17;7(24):2002274. doi: 10.1002/advs.202002274 (PMC7740085; doi:10.1002/advs.202002274)
Supplement: Supplementary file 1 — Supporting Information [file ADVS-7-2002274-s001.pdf]

WILEY-VCH

© 2020 Wiley-VCH GmbH

ADVANCED SCIENCE
Open Access

Supporting Information

for *Adv. Sci.*, DOI: 10.1002/advs.202002274

Ultraviolet Plasmons Empowered Photodetector of Ultrahigh Responsivity, Detectivity, and Broad Bandwidth Using Single-crystalline Aluminum Nanoholes array

Abhishek Dubey, Ragini Mishra, Yu-Hung Hsieh, Chang Wei Cheng, Bao-Hsien Wu, Lih-Juann Chen, Shangjr Gwo, Ta-Jen Yen**

WILEY-VCH
© 2020 Wiley-VCH GmbH

Supporting Information

Ultraviolet plasmons empowered photodetector of ultrahigh responsivity, detectivity, and broad bandwidth using single-crystalline aluminum nanoholes array

Abhishek Dubey[1], Ragini Mishra[2], Yu-Hung Hsieh[2,4], Chang Wei Cheng[3], Bao-Hsien Wu[1], Lih-Juann Chen[1], Shangjr Gwo[2,3,4], Ta-Jen Yen*[1]*

[1]Department of Material Sciences and Engineering, National Tsing Hua University,

Hsinchu 300, Taiwan R.O.C.

[2]Institute of Nanoengineering and Microsystem, National Tsing Hua University,

Hsinchu 300, Taiwan R.O.C.

[3]Department of Physics, National Tsing Hua University,

Hsinchu 300, Taiwan R.O.C.

[4]Research Centre for Applied Science, Academia Sinica,

Taipei 115-29, Taiwan R.O.C.

Corresponding Author

Email: *gwo@phys.nthu.edu.tw and *tjyen@mx.nthu.edu.tw

Keywords: UV Plasmonics, aluminum film, GaN, UV photodetection

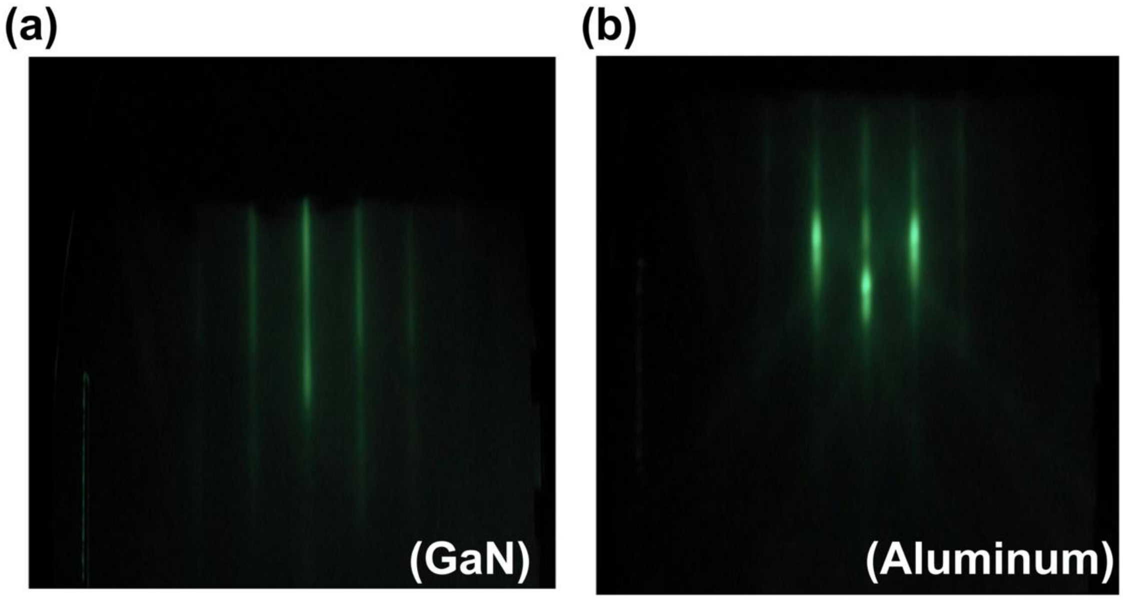

Figure S1. In-situ Analysis, Reflection high energy electron diffraction (RHEED) pattern **(a)** RHEED pattern of GaN before depositing single-crystalline Al film. **(b)** RHEED pattern of single-crystalline Al film, streaky RHEED pattern claims single-crystalline growth of Al film on GaN substrate.

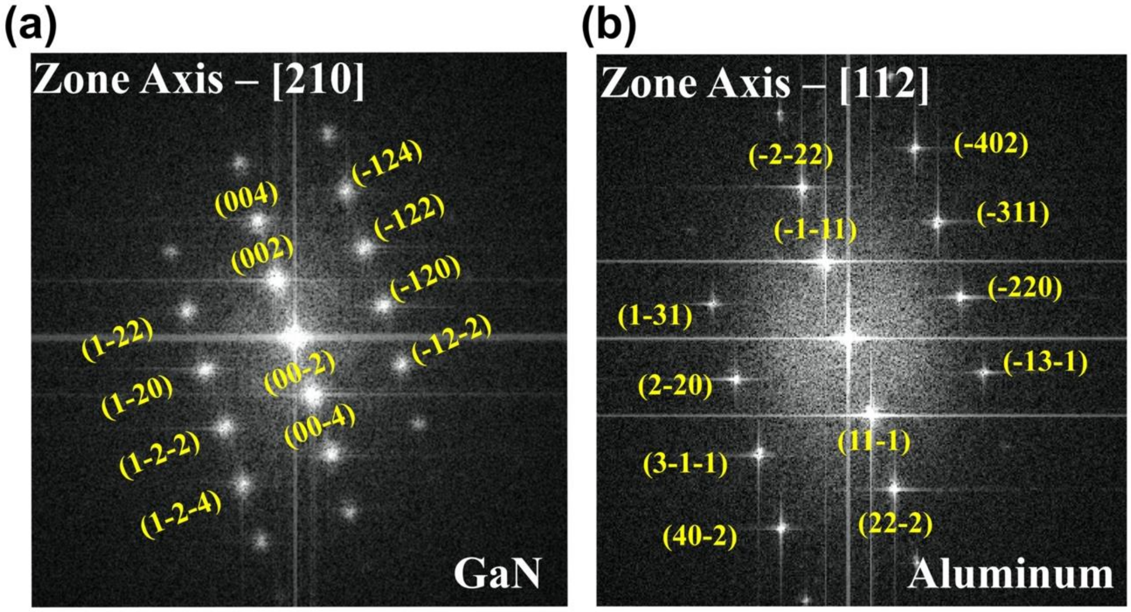

Figure S2. (a) & (b) Selective area electron diffraction (SAED) pattern of u-GaN/Al_2O_3 and single-crystalline Al film.

Thermionic emission model

$$I = AA^{*}T^{2}\ exp^{\frac{-q\emptyset_b}{kT}}$$

Where, A is effective Richardson constant, A* is area, T is absolute temperature, k is Boltzmann constant, q is electron charge and $\emptyset_b$ is Schottky barrier height in eV.

Al nanoholes thickness optimization using FDTD method.

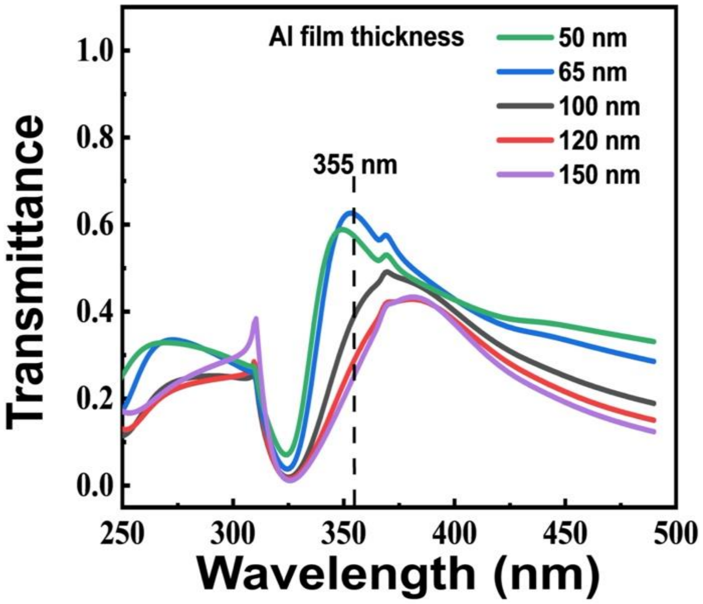

Figure S3. Extraordinary transmittance (EOT) spectra at Al nanoholes and GaN interface with various thickness of Al film (50 nm, 65 nm, 100 nm, 120 nm, 150 nm) using FDTD simulation method.

Transient Photocurrent Analysis

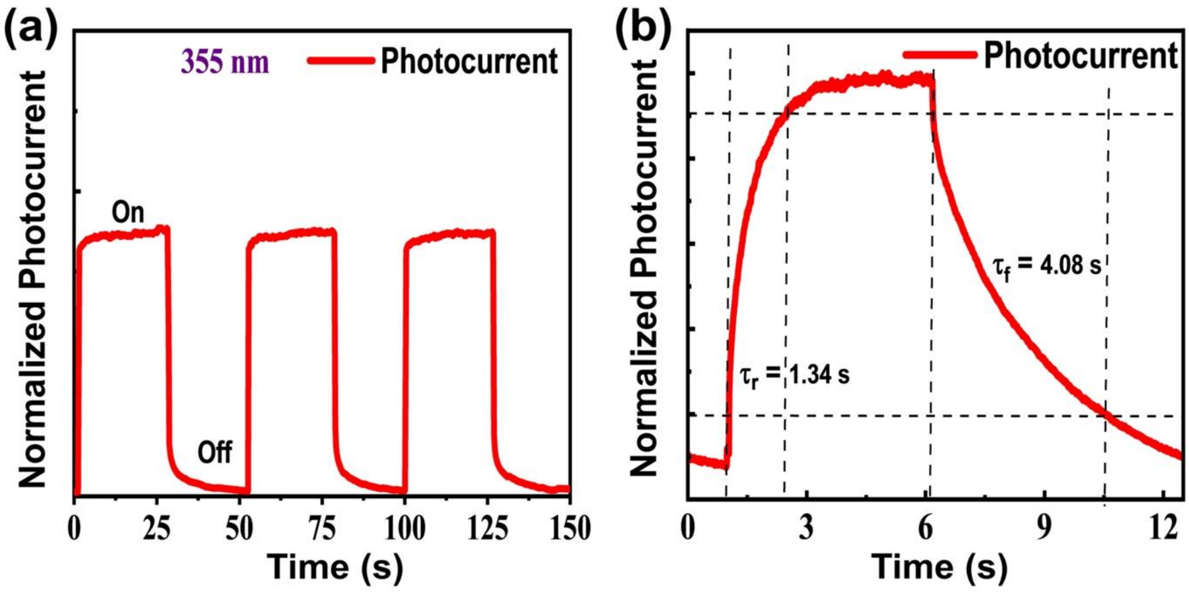

Figure S4. (a) Transient photocurrent measuremnet at 5V reverse bias using 355 nm laser excitation shows the stable On/Off behavior of photodetector using Al nanohole array. (b) Calculation of rising and falling time of GaN photodetector without Al nanhole array for 355 nm laser excitation at 5V reverse bias. 1.34 s and 4.08 s, rising and falling time are calculated.

Photodetection behavior for 325 nm laser excitation

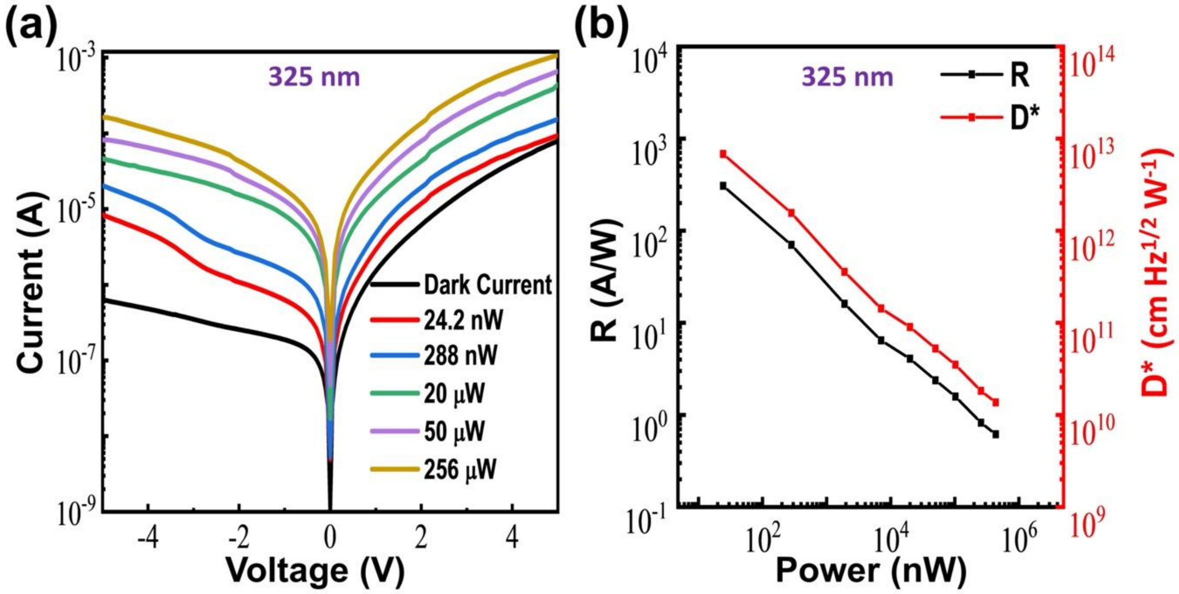

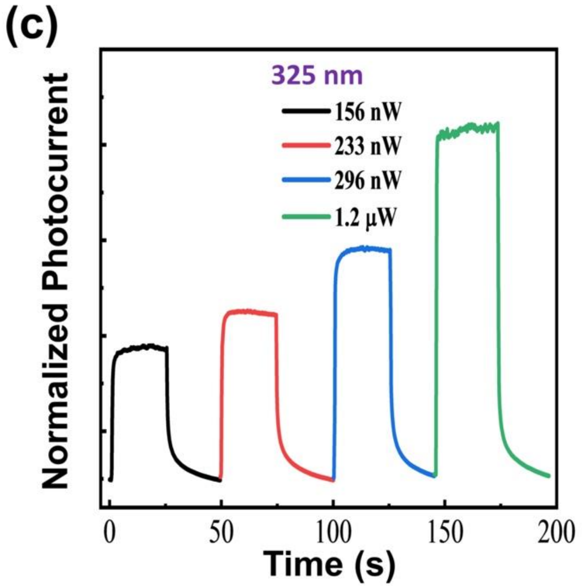

Figure S5. 325 nm photoresponse characteristics (a) I-V measurement of UV plasmons empowered photodetector in dark and using 325 nm laser with different illuminated optical power (24.2 nW, 288 nW, 20 μW, 50 μW and 256 μW). **(b)** Calculated responsivity (R) and detectivity (D*) as function of 325 nm laser illuminated power. The maximum detectivity and responsivity 7×10^{12} cm $Hz^{1/2}$ W^{-1} and 307 A/W are achieved at 5V reverse bias. **(c)** Transient photocurrent measurement using 325 nm laser with different illuminated optical power (156 nW, 233 nW, 296 nW and 1.2 μW).

Photodetection behavior for 266 nm laser excitation

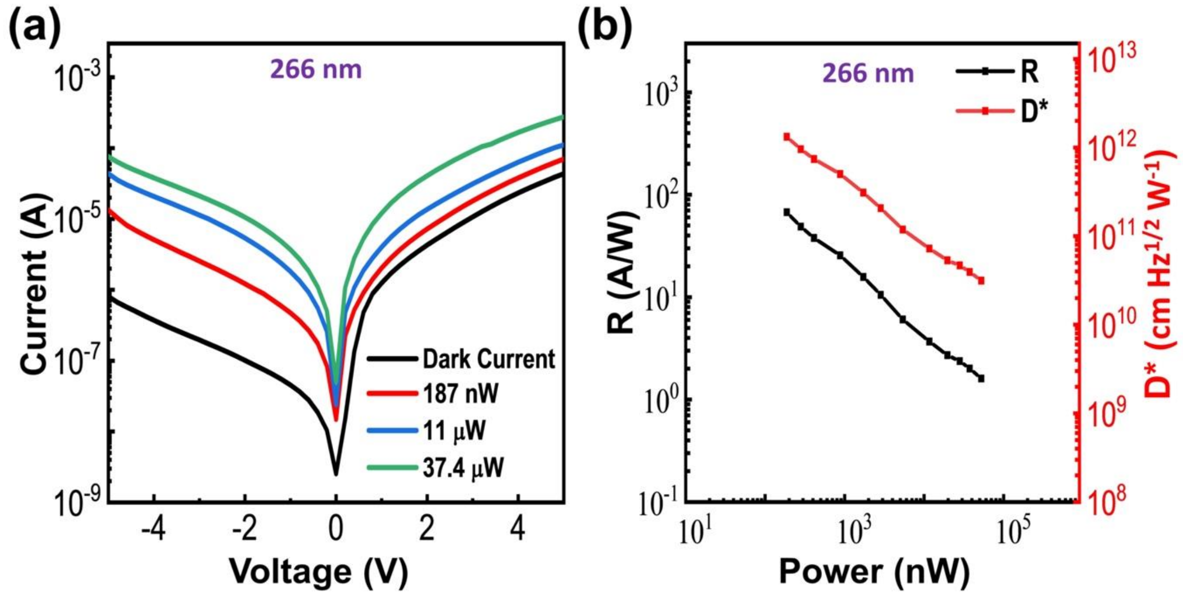

Figure S6. 266 nm photoresponse characteristics (a) I-V measurement of UV plasmons empowered photodetector in dark and using 266 nm laser with different illuminated optical power (187 nW, 11 µW, 37.4 µW). **(b)** Calculated responsivity (R) and detectivity (D*) as function of 266 nm laser illuminated power. The maximum detectivity and responsivity 1.32 × 10^{12} cm $Hz^{1/2}$ W^{-1} and 67.2 A/W are achieved at 5V reverse bias.
